# MiR-184 regulates insulin secretion through repression of Slc25a22

**DOI:** 10.7717/peerj.162

**Published:** 2013-09-24

**Authors:** Sumiyo Morita, Takuro Horii, Mika Kimura, Izuho Hatada

**Affiliations:** Laboratory of Genome Science, Biosignal Genome Resource Center, Institute for Molecular and Cellular Regulation, Gunma University, Japan

**Keywords:** miRNA, Insulin, Diabetes

## Abstract

Insulin secretion from pancreatic β-cells plays an essential role in blood glucose homeostasis and type 2 diabetes. Many genes are involved in the secretion of insulin and most of these genes can be targeted by microRNAs (miRNAs). However, the role of miRNAs in insulin secretion and type 2 diabetes has not been exhaustively studied. The expression miR-184, a miRNA enriched in pancreatic islets, negatively correlates with insulin secretion, suggesting that it is a good candidate for miRNA-mediated regulation of insulin secretion. Here we report that miR-184 inhibits insulin secretion in the MIN6 pancreatic β-cell line through the repression of its target Slc25a22, a mitochondrial glutamate carrier. Our study provides new insight into the regulation of insulin secretion by glutamate transport in mitochondria.

## Introduction

Pancreatic β cells play an important role in glucose homeostasis by secreting insulin. Insulin is released in response to elevated blood concentrations of glucose, and stimulates the cells in the liver, skeletal muscles, and fat tissue to absorb glucose from the blood. Secretion of an inappropriate amount of insulin, as a result of β cells dysfunction, leads to diabetes.

MicroRNAs are small non-coding RNAs that function as specific regulators of gene expression by targeting the 3′ untranslated region (3′ UTR) of the mRNA sequence, which results in mRNA degradation, mRNA deadenylation and translational repression (Bartel, 2004). MiRNAs have been exhaustively studied in cancer research and are considered to be particularly valuable as diagnostic markers and targets for gene therapy. By contrast, only a few studies have examined the role of these RNAs in insulin secretion and type 2 diabetes. Recently, some microRNAs were shown to have a functional role in β-cell development and function (Guay et al., 2012), and the regulation of insulin secretion.

Mir-184 is enriched in pancreatic islets and its expression is negatively correlated to insulin secretion (Bolmeson et al., 2011). Therefore, miR-184 is a good candidate for a miRNA-mediated regulation of insulin secretion. Here we report that miR-184 inhibits insulin secretion in the MIN6 islet β-cell line through the repression of its target Slc25a22, a mitochondrial glutamate carrier (Maechler, 2013).

## Materials and Methods

### Cell culture

MIN6 cells, a well differentiated mouse insulinoma β-cell line and display characteristics of pancreatic beta-cells, including insulin secretion in response to glucose (Miyazaki et al., 1990) (passages 24–28) were grown in Dulbecco’s modified Eagle’s medium containing high glucose (Sigma, St. Louis, MO, USA), 10% fetal bovine serum, 70 µM β-mercaptoethanol, and the cells were maintained in a humidified incubator with 95% air and 5% CO_2_ at 37°C.

### Prediction of miR-184 targets

miRanda, miRDB, miRwalk, RNA22, and TargetScanMouse software were used to retrieve potential targets of miR-184.

### Transfection of cells with miRNAs

Transient transfections of MIN6 cells with miRNAs were performed using Lipofectamine 2000 (Invitrogen), according to the manufacturer’s instruction. Briefly, cells were seeded in a 24-well tissue culture dish and transfected with 20 pmol of miRNA or siRNA on the same day. Cells were harvested 48–72 h after transfection. Transfected miRNAs were microRNA control Non-target RNA (cat. no. S10CM-0600, Cosmo Bio, Japan) and mmu-mir-184 (Cosmo Bio, Japan). The sequence of mmu-mir-184 is as follows: Sence: uggacggagaacugauaagggu, Anti-sence: ccuuaucaguucuccguccagc. Transfected siRNAs were Allstars Negative Control siRNA (cat. no. 1027280, Qiagen) and Gene Solution siRNA (Mm_Slc25a22_2, cat. no. SI01421238, Mm_Slc25a22_4, cat. no. SI01421252, Qiagen). The target sequences of siRNA as follows:

Mm_Slc25a22_2: cacccagaattatttattgaa, Mm_Slc25a22_4: ctgctgctgcttacagaatta.

### Plasmid construction

DNA fragment containing the 3′-UTRs of Slc25a22 (Accession; BC050887) was amplified by PCR using primers containing XbaI or FseI restriction sites. Amplified fragments were cloned into the corresponding sites in the 3′-UTR of the luciferase reporter vector, pGL3-Control (Promega, Madison, WI, USA). The primer sequences were as follows:

5′-GCTCTAGAGGTCCTGAAGGGACAACAAA-3′, 5′-GGGGCCGGCCGAAGGGCTGCTTTGATTCTG-3′.

### Reporter assay

Luciferase reporter plasmids were transiently transfected into MIN6 cells using Lipofectamine 2000 (Invitrogen, Grand Island, NY, USA), according to the manufacturer’s protocol. Briefly, cells seeded into a 24-well tissue culture dish were exposed to transfection mixtures containing 0.1 µg of luciferase reporter plasmid, 0.05 µg of pRL-TK control vector (Promega) and 10 pmol of miRNA. Cells were harvested 48 h after transfection. Luciferase assays were performed according to the manufacturer’s protocol (Promega). The pRL-TK plasmid was used to normalize firefly luciferase activity to Renilla luciferase activity, and to correct for transfection efficiency. Transfected miRNAs were microRNA control Non-target RNA (Cosmo Bio, Japan) and mmu-mir-184 (Cosmo Bio, Japan).

### Quantitative RT-PCR analysis

Total RNA was prepared from cells using Trizol (Invitrogen). Gene expression levels were measured with LightCycler 480 (Roche) using the SYBR Premix Ex Taq (TAKARA) according to the manufacturer’s instructions.

The real-time PCR was performed using the following cycle parameters: initial enzyme activation at 95°C for 5 s; followed by 45 cycles of 95°C for 5 s, 60°C for 20 s. Data was analyzed with the LightCycler 480 software (Roche), determining the threshold cycle (Ct) by the second derivative max method. The efficiency of PCR reaction shows 1.85–1.98 in the LightCycler 480 software.

Primers were designed with Primer3 (version 4.0). Melting curve PCR analysis was performed on all primers and rule out nonspecific amplification. All primers were verified with the real-time PCR analysis. Primer sequences were as follows:

Slc25a22 (Accession; BC050887): 5′-GAATTTTCCGTGCCCATGT-3′, 5′-CATGAGGCTAAGACCCTCCA-3′

Tcf7l2 (Accession; NM_001142922): 5′-GATGACCTAGGCGCTAACGA-3′, 5′-ATTCATTGACCAGCGAGGAC-3′

18s: 5′-CCCGAAGCGTTTACTTTGAA-3′, 5′-CCCTCTTAATCATGGCCTCA-3′.

### Insulin secretion assay

MIN6 cells were plated into 24-well plates at 1 × 10^5^ cells/well and grown for 2 days. Measurement of insulin secretion was accomplished by replacing the culture medium with modified KRB buffer (136 mM NaCl, 4.8 mM KCl, 2.5 mM CaCl_2_, 1.2 mM MgSO_4_, 1.2 mM KH_2_PO_4_, 5 mM NaHCO_3_, 10 mM HEPES, 0.1% BSA, pH 7.4). After a 1 h equilibration period at 37°C, cells were then incubated with KRB containing different glucose concentrations (0 mM, 25 mM) for 1 h. After 1 h, the supernatant was collected and the insulin content was measured using a mouse insulin ELISA kit (AKRIN-011T; Shibayagi, Japan). Cells were then lysed with Buffer RLT Plus lysis buffer (Qiagen) to collect RNA to analyze the expression of Slc25a22, and DNA to normalize the secreted insulin using the Allprep DNA/RNA mini kit (Qiagen). We extracted DNA from the same cells analyzed insulin secretion and use the amount of genomic DNA for normalization of cell number. Therefore, ng/ml/ng DNA means that the concentration of insulin in the medium (ng/ml) is normalized by the amount of genomic DNA.

### Statistical analysis

Student’s t test and ANOVA, Dunnett’s post hoc test for multiple comparison, was performed to ascertain statistical significance between the samples.

## Results

### MiR-184 inhibits glucose-induced insulin secretion in a pancreatic β-cell line

**Figure 1:**
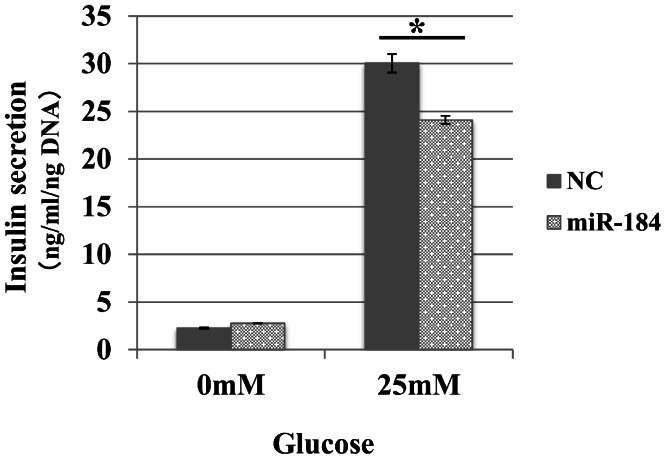
MiR-184 inhibits glucose-induced insulin secretion in the MIN6 islet β-cell line. MiR-184 or control miRNA were transfected into the MIN6 islet β-cell line and insulin secretion was examined. MiR-184 significantly inhibited glucose-induced insulin secretion in the MIN6 islet β-cell line. Data show the mean + SD, for *n* = 3 repeats. *p < 0.05, compared with control miRNA.

We examined whether miR-184 regulates insulin secretion in pancreatic β-cells because miR-184 is abundant in pancreatic islets and its expression is negatively correlated with insulin secretion (Bolmeson et al., 2011). We transfected miR-184 or control miRNA into the MIN6 islet β-cell line and found that miR-184 significantly inhibited glucose-induced insulin secretion in the MIN6 islet β-cell line (Fig. 1).

### Identification of targets of miR-184

**Figure 2:**
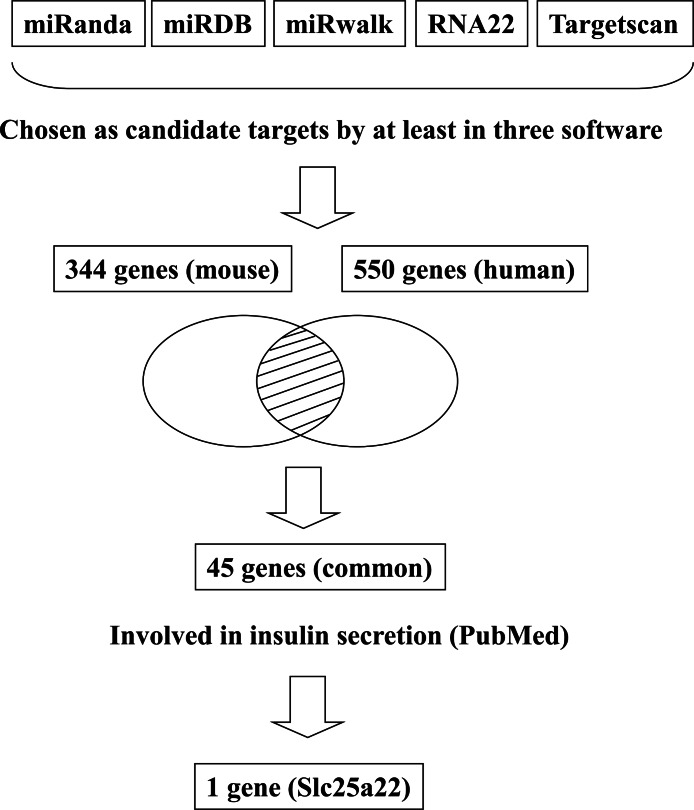
Flowchart for the selection of candidate targets of miR-184. Candidate targets were identified using the target prediction software miRanda, miRDB, miRwalk, RNA22 and TargetScan. The genes identified as targets by at least three of these software were selected. Among the genes selected, only the genes that were common to both mouse and human were subjected to further analysis. A search for these genes in the PubMed database revealed that only Slc25a22 was associated with insulin secretion.

**Figure 3:**
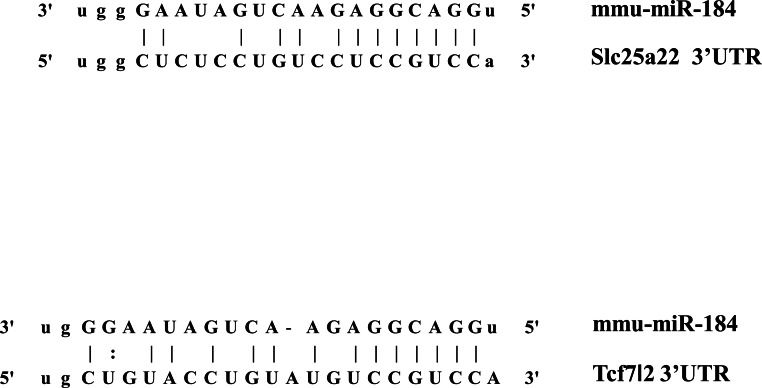
The candidate miR-184 target sites in the 3′-UTR of Slc25a22 and Tcf7l2.

In addition to inhibiting glucose-induced insulin secretion (Fig. 1), MiR-184 might also inhibit target genes involved in insulin secretion. Using at least three target prediction programs, we identified 344 mouse genes and 550 human candidate gene targets of miR-184 in miRanda (http://www.microrna.org/microrna/home.do), miRDB (http://mirdb.org/miRDB/), miRwalk (http://www.umm.uni-heidelberg.de/apps/zmf/mirwalk/), RNA22 (http://cbcsrv.watson.ibm.com/rna22.html) and TargetScan (http://www.targetscan.org/) (Fig. 2, Table S1). Among these genes, only 45 genes were conserved in mouse and human. A PubMed (http://www.ncbi.nlm.nih.gov/pubmed?holding=ijpgumlib) search revealed that only Slc25a22 is associated with insulin secretion (Casimir et al., 2009). The miR-184 target prediction site in Slc25a22 3′ UTR is shown in Fig. 3. Another target, Tcf7l2 (Fig. 3), is also involved in insulin secretion (Loder et al., 2008) and genetic variation in this gene is a risk factor for type 2 diabetes (Grant et al., 2006). However, this gene is a candidate target only in mouse and not in human.

### The miR-184 targets and regulates the expression of Slc25a22

**Figure 4:**
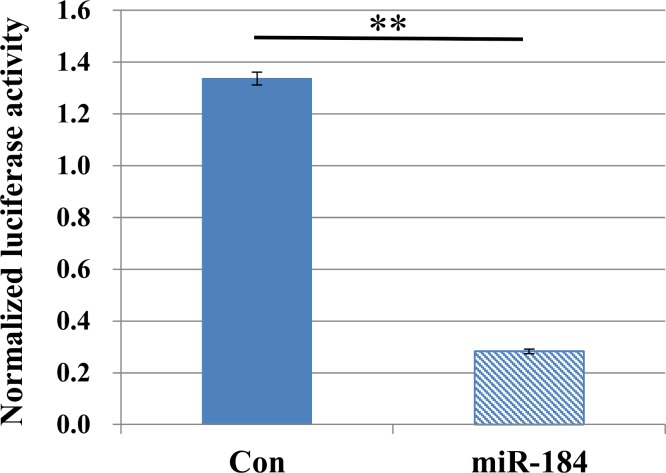
MiR-184 targets the 3′-UTRs of Slc25a22. Firefly luciferase activity of the Slc25a22 construct was measured 48 h after cotransfection of MIN6 cells with miR-184 or control miRNA. For each construct and cell line, data are normalized to the activity of Renilla luciferase to correct for transfection efficiency. Data show the mean + SD for *n* = 3 repeats. **p < 0.01, compared with control miRNA.

**Figure 5:**
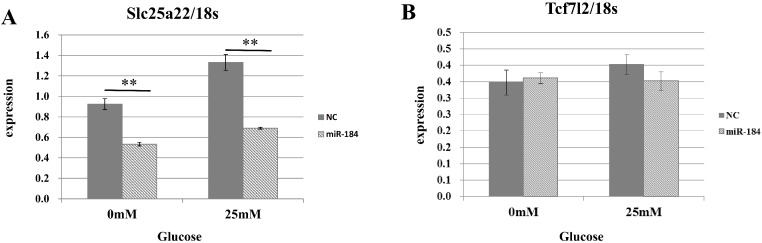
The effect of MiR-184 on endogenous Slc25a22. (A) Expression of Slc25a22 mRNA. Quantitative RT-PCR analyses of the expression levels of Slc25a22 mRNA were performed 48 h after transfection of MIN6 cells with miR-184 or control miRNA. The 18S ribosomal RNA was used to normalize the expression levels. Data show the mean + SD for *n* = 3 repeats. **p < 0.01, compared with control miRNA. (B) Expression of Tcf7l2 mRNA. Quantitative RT-PCR analyses of the expression levels of Tcf7l2 mRNA were performed 48 h after transfection of MIN6 cells with miR-184 or control miRNA. The 18S ribosomal RNA was used to normalize the expression levels. Data show the mean + SD for *n* = 3 repeats.

To validate the predicted interactions between Slc25a22 and miR-184, chimeric constructs in which the Slc25a22 3′-UTR was inserted into the 3′-UTR of the firefly luciferase gene were generated, and the chimeric constructs were cotransfected with miR-184 or control miRNA into MIN6 cells. We found ∼70% reduction in the luciferase signal (Fig. 4), suggesting an interaction between miR-184 and the Slc25a22 3′-UTR. To investigate whether ectopic expression of miR-184 downregulates the endogenous expression of Slc25a22 mRNA, quantitative RT-PCR analysis of RNA extracted from MIN6 cells transfected with miR-184 or control miRNA was performed. Compared to the control miRNA, transfection of cells with miR-184 resulted in reduced expression of endogenous Slc25a22 (Fig. 5A). Conversely, the expression of Tcf7l2, the only candidate target in mouse, was not downregulated in miR-184 transfected cells (Fig. 5B).

### miR-184 regulates insulin secretion through repression of Slc25a22

**Figure 6:**
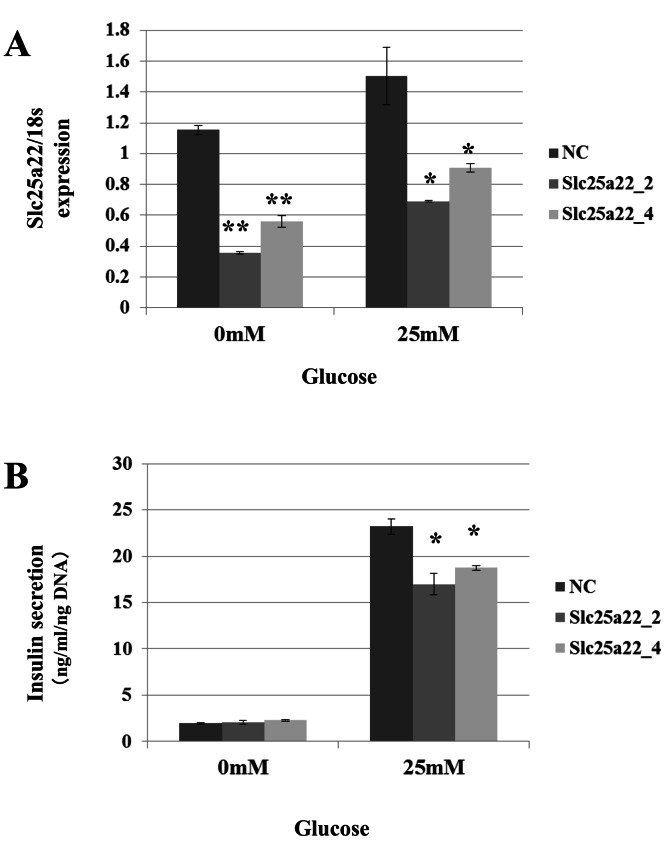
SiRNAs for Slc25a22 inhibits glucose-induced insulin secretion in the MIN6 islet β-cell line. SiRNA for Slc25a22 (Slc25a22_2, Slc25a22_4) or control siRNA was transfected into the MIN6 islet β-cell line. (A) Expression of Slc25a22 mRNA. Quantitative RT-PCR analyses of the expression levels of Slc25a22 mRNA were performed 48 h after transfection of MIN6 cells with Slc25a22 (Slc25a22_2, Slc25a22_4) or control siRNA. The 18S ribosomal RNA was used to normalize the expression levels. Data show the mean + SD for *n* = 3 repeats. **p < 0.01, (B) Insulin secretion assay. SiRNA for Slc25a22 significantly inhibited glucose-induced insulin secretion in the MIN6 islet β-cell line. Data show the mean + SD for *n* = 3 repeats. *p < 0.05, compared with control siRNA.

Targeting of Slc25a22 by miR-184 suggests that it contributes to the regulation of insulin secretion via the repression of Slc25a22. Indeed, miR-184 reduced the expression of Slc25a22 in the MIN6 islet β-cell line (Fig. 5A). In line with these findings, a previous study reported that Slc25a22 is associated with insulin secretion (Casimir et al., 2009). To address whether miR-184 regulates insulin secretion via the repression of Slc25a22, MIN6 cells were transfected with siRNA for Slc25a22 or control siRNA, and the insulin secretion levels were measured 48 h after transfection (Fig. 6). Compared with the control siRNA, siRNA directed against Slc25a22 significantly reduced the level of the expression of Slc25a22 (Fig. 6A), and glucose-induced insulin secretion (Fig. 6B) in MIN6 cells.

## Discussion

miR-184 is enriched in pancreatic islets and its expression negatively correlates with insulin secretion (Bolmeson et al., 2011). Here we found that miR-184 inhibits insulin secretion in a pancreatic β-cell line by repressing its target, Slc25a22. The SLC25 carrier family mediates solute transport across the inner mitochondrial membrane, a process that is poorly characterized, both with respect to the mechanisms and proteins involved in this process (Maechler, 2013). The mitochondrial glutamate carrier Slc25a22 is expressed in insulin-secreting β-cells and localized to the inner mitochondrial membrane (Maechler, 2013). Upon glucose stimulation, glutamate is produced from α-ketoglutarate (αKG) by glutamate dehydrogenase (GDH) and exported out of the mitochondria through the glutamate carrier, Slc25a22. Cytosolic glutamate targets the insulin granules because secretory vesicles require glutamate uptake for insulin exocytosis (Maechler & Wollheim, 1999; Høy et al., 2002; Eto et al., 2003; Gammelsaeter et al., 2011; Storto et al., 2006). When β-cells are forced to express an enzyme that decarboxylates intracellular glutamate, the glucose-induced rise in glutamate and the secretory response are impaired (Rubi et al., 2001). Abrogation of the mitochondrial enzyme GDH, which plays a key role in glucose-induced glutamate generation from the TCA cycle intermediate α-ketoglutarate (Vetterli et al., 2012), specifically in the β-cells of GDH knockout mice, reduces the secretory response (Carobbio et al., 2009). Slc25a22 plays an important role in the export of the newly synthesized glutamate out of mitochondria. We found that silencing of Slc25a22 reduces glucose-stimulated insulin secretion in MIN6 islet β-cells, as previously shown in INS-1E cells (Casimir et al., 2009). Prevention of glutamate release from β-cells results in a concomitant elevation of intracellular glutamate levels and glucose-induced insulin secretion (Feldmann et al., 2011). Finally, the repression of Slc25a22 expression by miR-184 reduces the level of cytosolic glutamate, which leads to reduced insulin secretory vesicle secretion.

## Supplemental Information

10.7717/peerj.162/supp-1 Table S1 Candidate target genes noted in Fig. 2
